# Recommendations for the Use of Serious Games in Neurodegenerative Disorders: 2016 Delphi Panel

**DOI:** 10.3389/fpsyg.2017.01243

**Published:** 2017-07-25

**Authors:** Valeria Manera, Grégory Ben-Sadoun, Teun Aalbers, Hovannes Agopyan, Florence Askenazy, Michel Benoit, David Bensamoun, Jérémy Bourgeois, Jonathan Bredin, Francois Bremond, Carlos Crispim-Junior, Renaud David, Bob De Schutter, Eric Ettore, Jennifer Fairchild, Pierre Foulon, Adam Gazzaley, Auriane Gros, Stéphanie Hun, Frank Knoefel, Marcel Olde Rikkert, Minh K. Phan Tran, Antonios Politis, Anne S. Rigaud, Guillaume Sacco, Sylvie Serret, Susanne Thümmler, Marie L. Welter, Philippe Robert

**Affiliations:** ^1^Université Côte d’Azur, Cognition, Behaviour, Technology – CoBTeK Nice, France; ^2^Université Côte d’Azur, INRIA, STARS Sophia Antipolis, France; ^3^Radboudumc Alzheimer Center, Donders Institute for Medical NeuroSciences, Radboudumc Nijmegen, Netherlands; ^4^IEM Rossetti des PEP 06 Nice, France; ^5^Centre Ressources Autisme, Service Universitaire de Psychiatrie de l’Enfant et de l’Adolescent, Children’s Hospitals of Nice CHU-Lenval Nice, France; ^6^IA Association Nice, France; ^7^Université Côte d’Azur, Centre Hospitalier Universitaire, Hôpital Pasteur Nice, France; ^8^Université Côte d’Azur, Centre Hospitalier Universitaire de Nice, Centre Mémoire de Ressource et de Recherche Nice, France; ^9^College for Education, Health and Society, Miami University, Oxford OH, United States; ^10^Department of Veterans Affairs, VA Palo Alto Health Care System, Livermore CA, United States; ^11^Department of Psychiatry and Behavioral Sciences, Stanford University School of Medicine, Stanford CA, United States; ^12^Genious Group Montpellier, France; ^13^Departments of Neurology and Psychiatry and Department of Physiology, University of California, San Francisco, San Francisco CA, United States; ^14^Bruyère Memory Program, Bruyère Research Institute Ottawa, ON, Canada; ^15^Department of Family Medicine, University of Ottawa Ottawa, ON, Canada; ^16^Department of Systems and Computer Engineering, Carleton University Ottawa, ON, Canada; ^17^Department of Geriatrics and Radboudumc Alzheimer Center, Radboud University Medical Center Nijmegen, Netherlands; ^18^1st Department of Psychiatry, Eginition Hospital, National and Kapodistrian University of Athens Athens, Greece; ^19^Hopital Broca, Assistance Publique-Hôpitaux de Paris Paris, France; ^20^Faculty of Medicine, Université Paris Descartes Paris, France; ^21^UMR-S975, Institut du Cerveau et de la Moelle épiniere, Université Pierre et Marie Curie Paris, France; ^22^U975, INSERM Paris, France; ^23^UMR 7225, CNRS Paris, France; ^24^Département de Neurologie, Hôpitaux Universitaires Pitié-Salpêtrière, Assistance Publique-Hôpitaux de Paris Paris, France

**Keywords:** serious games, neurodegenerative disorders, recommendations, ICT, Delphi Technique

## Abstract

The use of Serious Games (SG) in the health domain is expanding. In the field of neurodegenerative disorders (ND) such as Alzheimer’s disease, SG are currently employed both to support and improve the assessment of different functional and cognitive abilities, and to provide alternative solutions for patients’ treatment, stimulation, and rehabilitation. As the field is quite young, recommendations on the use of SG in people with ND are still rare. In 2014 we proposed some initial recommendations (Robert et al., 2014). The aim of the present work was to update them, thanks to opinions gathered by experts in the field during an expert Delphi panel. Results confirmed that SG are adapted to elderly people with mild cognitive impairment (MCI) and dementia, and can be employed for several purposes, including assessment, stimulation, and improving wellbeing, with some differences depending on the population (e.g., physical stimulation may be better suited for people with MCI). SG are more adapted for use with trained caregivers (both at home and in clinical settings), with a frequency ranging from 2 to 4 times a week. Importantly, the target of SG, their frequency of use and the context in which they are played depend on the SG typology (e.g., Exergame, cognitive game), and should be *personalized* with the help of a clinician.

## Introduction

The use of Information and Communication Technologies (ICT) in the health domain is progressively expanding. Recently, increasing attention is devoted to the field of neurodegenerative disorders (ND), such as Alzheimer’s disease (AD), where ICT is employed both to support and improve the assessment of different functional and cognitive abilities (Aalbers et al., 2013; Robert et al., 2013; König et al., 2015), and to provide alternative solutions for patients’ treatments, stimulation, and rehabilitation. A field which is rapidly growing is that of Serious Games (SG), which are mental and/or physical contests played with a computer in accordance with specific rules, which use entertainment to promote training, education, health, public policy, and strategic communication objectives (Zyda, 2005). Contrary to other ICT-based tools, such as computerized testing or cognitive training, SG embed the playful and entertaining aspects typical of video-games into the ‘serious’ activity, by applying a pedagogic scenario to the game scenario (Alvarez, 2007). The features typical of SG, such as the presence of a game challenge and of long-term goals, have been proposed to make SG more adapted than classical computer-based training to sustain generalization of learnt activities to real life situations (Whyte et al., 2015). For this reason, recommendations for the *design* of SG targeting ND are starting to emerge (e.g., Bouchard et al., 2012; Fua et al., 2013; Mader, 2015). However, recommendations on the *use* of SG in these populations are still rare. In 2014, we proposed some initial recommendations for the use of SG in people with ND, gathered by experts in the field during a consensus group (Robert et al., 2014). Specifically, we systematically analyzed the Strengths, Weaknesses, Opportunities and Threats (SWOT) of employing SG with these patients, and reported practical guidelines on when, where, and with whom SG should be employed, and to specify which categories of patients and which abilities should be targeted. Since then, a few empirical studies were published on the use of SG in these populations, describing the feasibility of employing SG targeting improvements in social/emotional wellbeing (Beneviste et al., 2012), SG training cognitive abilities such as executive functions (Manera et al., 2015), and Serious Exergames including a combination of cognitive training and physical training (Ben-Sadoun et al., 2016). A few more SG were designed for these patients to train cognitive abilities such as memory (Kim et al., 2015) and several aspects of visual attention (Mader et al., 2012), but they have not been tested so far on people with ND. Based on these new works and on the experience gained by different research centers involved in the use of SG in people with ND in the last years (e.g., the CoBTeK research laboratory of the Université Côte d’Azur, France; the Hopital Broca in Paris, France; the Radboud Alzheimer Center, Nijmegen, the Netherlands; Neuroscape in San Francisco, CA, United States) the aim of the present work is to update the recommendations published in 2014, thanks to a Delphi expert panel.

### Neurodegenerative Disorders (ND)

ND progress through several stages in several years, and ultimately lead to dementia, a decline in mental ability severe enough to interfere with activities of daily living. Dementia can result from different causes, the most common being AD. It is often preceded by a pre-dementia stage, known as mild cognitive impairment (MCI), characterized by a cognitive decline greater than expected for an individual’s age, which, however, does not interfere notably with activities of daily living (Petersen et al., 1997). Depending on the etiology and the disease’s stage, dementia can be characterized by cognitive, behavioral, motor, and/or functional symptoms. The biological processes involved in ND are very heterogeneous, and include neuroinflammation, gliosis, synaptic loss, neurodegeneration, cerebral atrophy, and alterations of the blood–brain barrier permeability (Raz et al., 2016). These molecular alterations are due, among others, to alterations in the bioenergy metabolism, to hypoperfusion/hypoxia, and to dysfunctions of the cerebrovascular hemodynamic. From a therapeutic point of view, much research aims to modify the course of the disease or to reduce the impact of the clinical symptoms. Social interaction, physical and cognitive activities, and motivation can have a major impact on the disease progression. Hence, non-pharmacological approaches targeting people’s lifestyle are of particular interest.

### Serious Games for People with Neurodegenerative Disorders

Boosted by the publication of a Nature letter showing that video game training can enhance cognitive control in older adults (Anguera et al., 2013), there is now a growing interest in developing SG specifically adapted to people with ND. Evidence is accumulating showing that video-games and VR-applications can successfully be employed for early detection and monitoring of physical and cognitive impairment (e.g., Tarnanas et al., 2013; Aalbers et al., 2016; Negut et al., 2016; Zygouris et al., 2017), but also to train physical and cognitive abilities in people with AD, MCI, and related disorders. In the field of training, most of the research work so far has been conducted employing commercial video-games and cognitive games (such as Wii Fit and Wii Sport, Lumosity) designed for an entertaining purpose, and with a ‘typical’ healthy user in mind. In their review on the use of video-games in people with dementia-related disorders, McCallum and Boletsis (2013) showed that: (a) Exergames, i.e., games that promote physical condition and/or aerobic fitness can positively affect several areas of mobility in participants with mild AD and MCI, such as balance and gait (Padala et al., 2012), and voluntary motor control (Legouverneur et al., 2011); (b) cognitive games can improve cognitive functions, such as attention and memory (Stavros et al., 2010; Weybright et al., 2010) and visuo-spatial abilities (Yamaguchi et al., 2011); (c) physical and cognitive games can have a positive impact on social and emotional functions, for instance they can improve the mood and increase positive affect and sociability (Weybright et al., 2010; Boulay et al., 2011) and reduce depression (Férnandez-Calvo et al., 2011). As the field is young, less evidence is available on the efficacy of SG specifically designed for the training of people with ND. However, evidence collected in three studies suggests that SG and Serious Exergames are acceptable and motivating even for people with dementia. Beneviste et al. (2012) designed a SG based on musico-therapy targeting patients with AD and mild to moderate dementia aiming at improving patients’ self-image and to reduce their behavioral symptoms. Players use Wiimotes with WiiPistol to improvise or play predefined songs on a virtual keyboard. Results of a 2-month usability study conducted on seven AD patients confirmed that the SG was usable by AD patients despite their motor and cognitive impairments, and that patients were overall very satisfied with the game and expressed a desire to repeat the experience. Manera et al. (2015) found similar results with ‘Kitchen and cooking,’ a SG designed to train executive functions and praxis in people with MCI and early AD. The results of a 4-week feasibility study conducted on 21 participants (with MCI or early to moderate AD) confirmed that the game was acceptable and usable both at home and in a nursing home setting, and that patients were able to improve their game performance over the training, as confirmed by the fact that they became faster in the game activities. Finally, Ben-Sadoun et al. (2016) showed that X-Torp, a Serious Exergame designed to train physical, cognitive and social functions, was well accepted by people with dementia and MCI (*N* = 10) and healthy elderly controls (*N* = 8), and that during a 1-month trial participants experienced mainly positive emotions, improved their cardio-respiratory fitness, and were able to progress in the cognitive games scenarios. A summary of the training features of these three feasibility/pilot studies in reported in **Table 1**.

**Table 1:** Summary of the existing studies on SG tested on participants with MCI and/or dementia.

	MinWii	Kitchen and cooking	X-Torp
Feasibility study	Beneviste et al., 2012	Manera et al., 2015	Ben-Sadoun et al., 2016

SG for whom?	Older adults with AD and mild to severe dementia	Older adults with MCI and mild to moderate dementia	Older adults with MCI and mild to moderate dementia

What is the clinical target?	Increase self-esteem; reduce behavioral symptoms	Train executive functions (e.g., planning) and praxis	Train physical and cognitive activity in a positive emotional context

Where was it used?	Clinical setting	Clinical setting, home, nursing home	Clinical setting

With whom was it used?	Clinician and by groups of 3–4 participants	Clinician and alone	Clinician

When (how frequently) was it used?	Once a week	Once a week in a clinical setting; As much as they wanted at home/nursing home	3 times/week

Training duration	4–8 weeks	4 weeks	5 weeks

Session duration	Mean of 10-20 min	As much as wanted	Mean 35–40 min

Number of participants	7	21 (MCI and ND)	18 (10 ND, 8 controls)

Participants’ clinical baseline data	MMSE between 10 and 25	For AD, MMSE between 15 and 24: for MCI, MMSE between 24 and 30	For ND, MMSE between 16 and 27, CDR > 0

**FIGURE 1:**
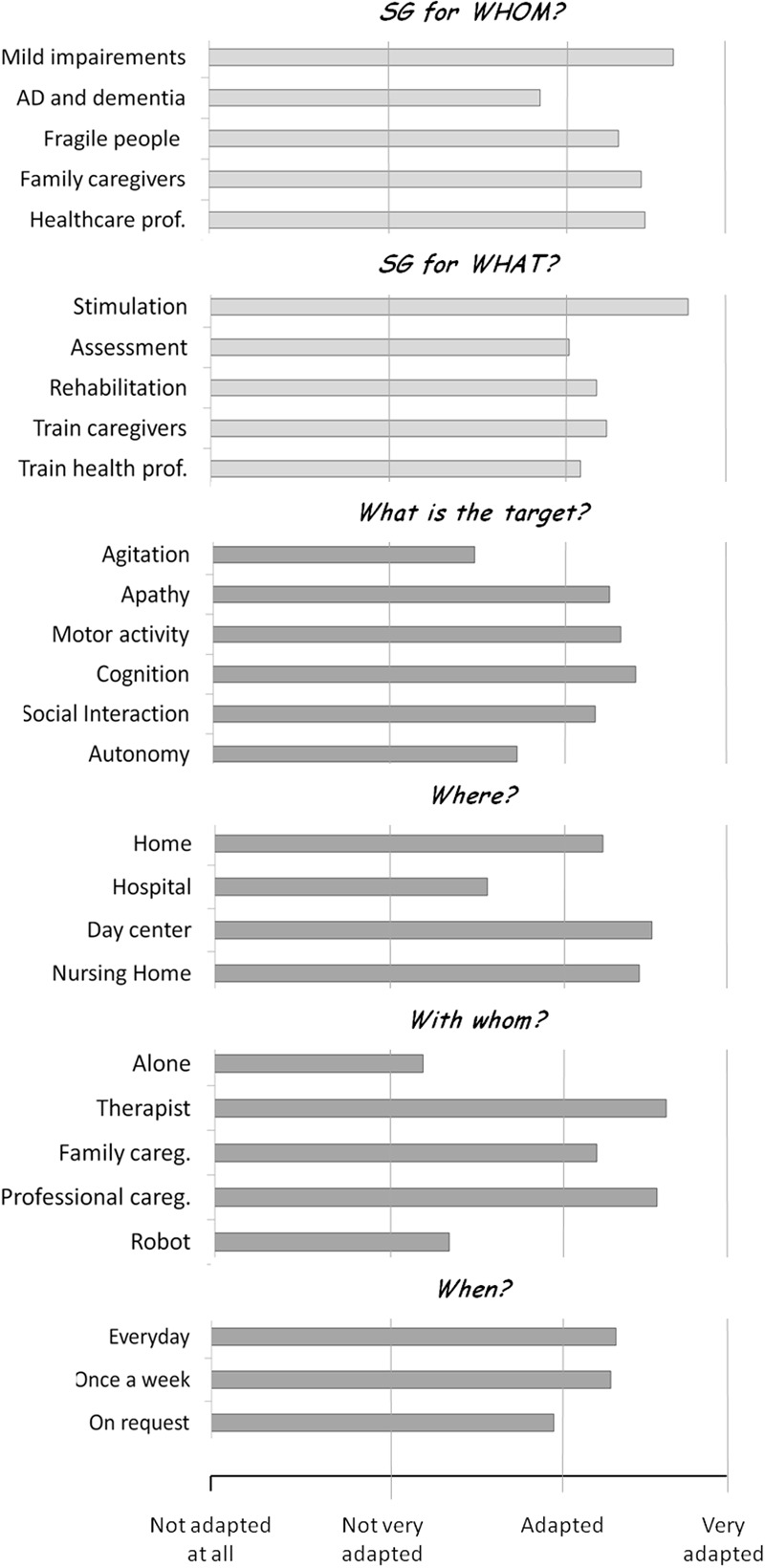
Results of the recommendations for the use of SG drafted in the 2013 IA workshop. Adapted from Robert et al. (2014). In a Delphi panel, participants were provided with questions concerning the ideal clinical population and target of SG (light gray bars). Next they were asked to focus on older adults with cognitive impairment, and were asked questions about the clinical target, the frequency of use, and the context of use (with whom and where) of SG in this population (dark gray bars). For each question, participants were provided with a number of response alternatives, and asked to rate each item on a 4-point scale, from ‘not adapted at all’ to ‘completely adapted.’

Data on the frequency of use employed in the three feasibility studies is converging with the recommendations reported by Robert et al. (2014). A consensus group including expert of ND (health domain) and of VG/SG design (ICT domain) and commercialization (business domain) met in a standalone meeting, and were asked to respond to questions concerning the ideal clinical population and target of SG, their frequency of use, and their context of use (with whom and where). Results (reported in **Figure 1**) suggested that: (a) SG were considered between ‘adapted’ and ‘very adapted’ to people with MCI, and between ‘not very adapted’ and ‘adapted’ to people with AD and related disorders; (b) SG was rated between ‘adapted’ and ‘very adapted’ for assessment, stimulation and rehabilitation of people with AD and related disorders, to train family caregivers and healthcare professionals, with the best rated target being stimulation; clinical targets rated between ‘adapted’ and ‘very adapted’ included physical, cognitive and social stimulation, as well as apathy (while agitation and improvements in activities of daily living were considered between ‘not very adapted’ and ‘adapted’); (c) SG should be employed regularly (‘everyday’ and ‘once a week’ were both rated between ‘adapted’ and ‘very adapted,’ while ‘on request’ was rated between ‘not very adapted’ and ‘adapted’); (d) SG were rated between ‘adapted’ and ‘very adapted’ to be employed at home, in day centers and nursing homes, and between ‘not very adapted’ and ‘adapted’ to be employed in the hospital; (e) SG were rated between ‘adapted’ and ‘very adapted’ to be employed with someone (being either a therapist, a family caregiver or a professional caregiver), and between ‘not very adapted’ and ‘adapted’ to be employed alone (or with a robot).

In the present paper we aimed to update and refine these initial recommendations thanks to a Delphi expert panel. A number of methodological changes were performed compared to the 2014 consensus panel. First of all, the 2014 recommendations were collected in a single round, without following the classical Delphi methodology. Indeed in the standard Delphi method (Linstone and Turoff, 1975) experts are asked to answer questions in two (or more) rounds. After each round, a facilitator provides a summary of the experts’ responses, and encourages the experts to analyze, comment and (eventually) revise their earlier answers in light of the commentaries of other members of the panel. The recommendations reported in the present paper followed the classical Delphi method (see below). Second, in 2014 we asked participants to rate each question on a 4-point scale (‘not adapted at all,’ ‘not very adapted,’ ‘adapted’ and ‘very adapted’). Here, we selected instead a 5-point scale (‘not adapted at all,’ ‘not very adapted,’ ‘adapted,’ ‘very adapted’ and ‘completely adapted’) to improve the symmetry of this Likert-type scale. Third, in the 2014 study several questions collapsed persons with MCI and more advanced stages of AD (‘people with AD and related disorders’). As recommendations for these two groups may be quite different, in the present study we kept them as separated categories for all the questions. Forth, we collapsed in a single questions the ‘Where’ and ‘With whom’ questions (see below), in order to better explore the exact contributions of (and interactions between) these two factors. Finally, we added a number of response alternatives to several questions, in order to obtain more precise information (e.g., in the ‘When’ question, we employed 6 response alternatives instead of 3).

## Methods

The recommendations reported in the present paper were collected and discussed during the workshop “Innovation Alzheimer 2016,” organized by the CoBTeK (Cognition – Behaviour – Technology) Research Unit of the Université Côte d’Azur, in Nice (France) on September 28th, 2016, in occasion of the 10th World Conference of Gerontechnology (ISG2016).

### Participants

The expert panel (*N* = 23) included researchers and health care professionals working on autism and other neurological and developmental disorders (*n* = 6), neurodegenerative disorders (*n* = 10), or both neurodevelopmental and neurodegenerative disorders (*n* = 2); ICT engineers (*n* = 2); and game developers (*n* = 3).

### Procedure

Following the DELPHI method, a list of questions was sent to all participants a week before the meeting via web-survey. Who, Where, When, and What questions were used as guidelines to structure the survey. Specifically, participants were asked to respond to the following questions:

(a) SG for *whom*? Are SG adapted (i.e., appropriate) to the following populations?
  • Mild Cognitive Impairment (MCI);
  • Early to moderate dementia due neurodegenerative disorders such as AD (dementia)
(b) *What* is the clinical target (in each condition)*?*
  • Assess (Physical, cognitive functions, IADL, …)
  • Train physical activity (Muscles, cardio-resp. fitness)
  • Train cognitive functions (Attention, memory, executive functions,…)
  • Improve autonomy (IADL)
  • Improve wellbeing (Increase positive emotions, self-esteem; reduce negative emotions, stress)
  • Favor social exchanges (Improve sociability and favor relations)
  • Teach contents
(c) *Where* should SG be used, and *with whom* (in each condition)?
  • At home, alone
  • In a clinical facility (e.g., hospital, long-term residence, at the doctor’s office), alone
  • At home, with a trained (professional or family) caregiver
  • In a clinical facility, with a trained (professional or family) caregiver
(d) *When* (how frequently) should SG be used (in each condition)?
  • Once a week
  • Twice a week
  • 3 times a week
  • 4 times a week
  • 5 times a week
  • Everyday

Participants were asked to rate each item on a 1–5 scale (1 = not adapted at all; 2 = not very adapted; 3 = adapted; 4 = very adapted; 5 = completely adapted).

## Data Analysis

Results were collected, and analyzed. During the workshop, a discussion session was organized with the objective to comment on the survey results, and to generate practical recommendations for the use of SG in MCI and dementia. Ratings from one participant were not considered for data analysis because more than 50% of responses were missing. Thus, reported data refer to 22 participants. For descriptive analysis purposes, we reported mean ratings. In order to compare ratings obtained for people with MCI and people with dementia in the first (“SG for whom?”) and second (“What is the clinical target?”) questions, we performed separate repeated-measures ANOVAs on each response item with Group (MCI vs. dementia) as within subject factor. For the second question, in order to account for multiple comparisons (*N* = 7), Bonferroni corrections were applied (α = 0.05/7 = 0.007). The third question (“*Where* should SG be used, and *with whom*”) was analyzed by means of a repeated-measures ANOVA with Group (MCI vs. dementia), Where (home vs. clinical facility) and with Whom (alone vs. with a trained caregiver) as within-subject factors, in order to analyze the effect of the three factors and their interactions. Finally, the fourth question (“*When* should SG be used?”) was analyzed by means of a repeated-measures ANOVA with Group (MCI vs. dementia) and Frequency (1, 2, 3, 4, 5 and 7 days per week) as within-subject factors. As the methodology employed in the present study is not completely comparable to that employed in the 2014 recommendations paper, we compared the results of the two studies only at a descriptive level.

## Results

### SG for Whom?

**FIGURE 2:**
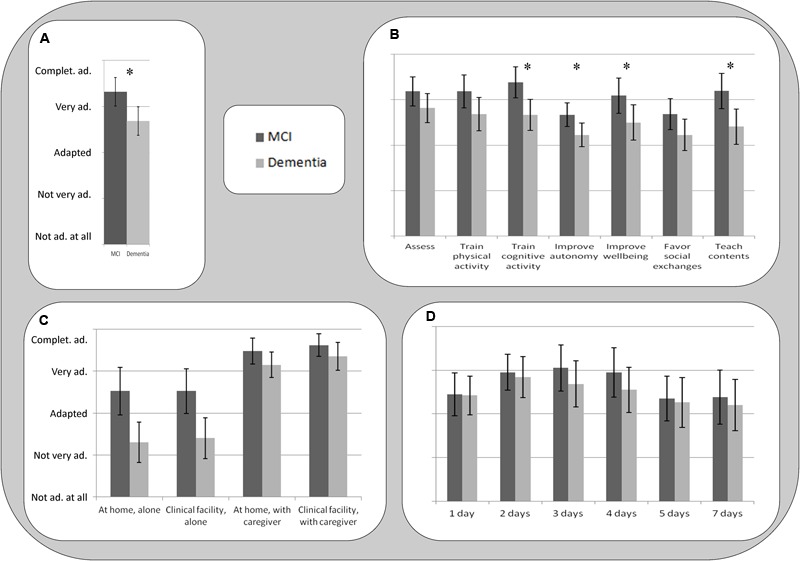
Results of the rating questions. Error bars represents 95% Confidence Intervals for the ANOVAs. ^∗^Reported in **(A,B)** show significant paired contrasts in the repeated-measures ANOVA with Group (MCI vs. Dementia) as within-subject factor. **(A)** SG for *whom*? Are SG adapted to older people: with MCI; and early to moderate dementia due neurodegenerative disorders (dementia); **(B)** What is the clinical target? Assess; train physical activity; train cognitive functions; improve autonomy; Improve wellbeing; favor social exchanges; teach contents. **(C)** *Where* should SG be used, and *with whom*? At home, alone; in a clinical facility, alone; at home, with a trained caregiver; in a clinical facility, with a trained caregiver. **(D)** *When* (how frequently) should SG be used? 1, 2, 3, 4, 5, and 7 days per week.

Results are reported in **Figure 2A**. SG were rated between ‘very adapted’ and ‘completely adapted’ for people with MCI, and between ‘adapted’ and ‘very adapted’ to people with dementia. Repeated-measures ANOVA confirmed that SG were rated as more adapted to people with MCI compared to people with dementia [*F*_(1,21)_ = 16.87, *p* = 0.001], suggesting that SG are considered as more adapted to people with initial cognitive decline than to people which are already loosing autonomy in activities of daily living. However, SG are considered to be adaptable also to people with dementia.

### What Is the Clinical Target?

Results are reported in **Figure 2B**. Participants reported that SG are between ‘very adapted’ and ‘completely adapted’ for assessment, to train physical and cognitive functions, improve wellbeing, and teach contents. Improving autonomy and favoring social exchanges were considered from ‘adapted’ to ‘very adapted.’ For people with dementia, SG were rated between ‘adapted’ and ‘very adapted’ for all the specified targets. This is in line with responses to the question ‘SG for whom,’ and it suggests that all these domains do represent useful targets for SG in this population. Repeated-measures ANOVAs (Bonferroni corrected) conducted to compare people with MCI and dementia for each category suggested that SG were considered as more adapted to people with MCI compared to people with dementia to train cognitive functions [*F*_(1,20)_ = 17.44, *p* < 0.001], to improve autonomy [*F*_(1,20)_ = 10.80, *p* = 0.004] and wellbeing [*F*_(1,21)_ = 9.32, *p* = 0.006] and to teach contents [*F*_(1,20)_ = 15.42, *p* = 0.001]. All the other contrasts did not reach statistical significance (*p* > 0.007).

### Where Should SG Be Used, and with Whom?

Results for all these patient populations (**Figure 2C**) suggest that SG are mostly adapted (between ‘very adapted’ and ‘completely adapted’) to be employed with a trained caregiver, both in a home and in a clinical setting. The use of SG by patients alone was rated between ‘adapted’ and ‘very adapted’ for people with MCI, and between ‘not very adapted’ and ‘adapted’ for people with dementia. Repeated-measures ANOVA with Group (MCI vs. dementia), Where (home vs. clinical facility) and With whom (alone vs. with a trained caregiver) as within-subject factors confirmed a significant effect of With whom factor [*F*_(1,19)_ = 54.82, *p* < 0.001], with SG use with a caregiver as more adapted compared to SG use alone. The Where factor was not statistically significant [*F*_(1,19)_ = 1.30, *p* = 0.269], thus suggesting that SG are considered as equally adapted to be employed at home and in a clinical facility. A significant effect of Group was also found, with SG rated as more adapted to be employed with people with MCI compared to people with dementia in all the settings [*F*_(1,19)_ = 22.03, *p* < 0.001]. Interestingly, a significant interaction between Group and With whom factor was also found [*F*_(1,19)_ = 12.67, *p* = 0.002], suggesting that employing SG with a trained caregiver is especially important for people with dementia. No other 2-way or 3-way interaction reached statistical significance (all *p*s > 0.360).

### When (How Frequently) Should SG Be Used?

Results (**Figure 2D**) suggest that all the game frequencies were rated between ‘adapted’ and ‘completely adapted’ for all conditions, and the mean ratings of the different game frequencies can be visually described as skewed Gaussian distributions. Repeated-measures ANOVA with Group (MCI vs. dementia) and Frequency (1, 2, 3, 4, 5 and 7 days per week) as within-subject factors revealed no significant main effect of Group [*F*_(1,18)_ = 3.45, *p* = 0.008], and Frequency [*F*_(5,90)_ = 1.72, *p* = 0.139], and no significant interaction between Group and Frequency [*F*_(5,90)_ = 0.60, *p* = 0.704]. Converging with descriptive data, the ANOVA’s contrast tests revealed an almost-significant quadratic contrast [*F*_(1,18)_ = 4.27, *p* = 0.053], suggesting that categories in the middle of the curve were rated as more adapted compared to extreme values (1 and 7 days per week). For participants with MCI and dementia, the highest scores were obtained for game frequencies from 2 to 4 days a week.

## Discussion

Since the publication of our previous recommendations on the use of SG (Robert et al., 2014), a number of SG were developed and tested with older people with MCI and dementia to train physical and cognitive abilities and to improve emotional wellbeing (Beneviste et al., 2012; Manera et al., 2015; Ben-Sadoun et al., 2016). These studies showed promising results, but also a number of usability challenges. Reported difficulties included, for instance, a higher fatigability of people with MCI and dementia in physically stimulating SG compared to healthy older adults (Ben-Sadoun et al., 2016), and, for several participants with cognitive impairment, low motivation to play SG when not accompanied by a family or professional caregiver (Manera et al., 2015). These difficulties were reported despite these SG were specifically designed for people in these populations. This suggests that the feasibility of employing SG with people with ND does not depend only on the game design features: an important component is the training format and structure. This confirms the importance of providing recommendations not only for the *design*, but also on the *use* of SG, that should be tested in clinical trials. Starting from the recommendations published in 2014 (Robert et al., 2014), the aim of the present paper was to draft guidelines for the use of SG in people with ND, thanks to a review of recently published studies employing SG in these populations, and gathering the opinion of experts in the field in a Delphi expert panel. A summary of the main recommendations is reported in **Table 2**.

**Table 2:** Recommendations for the use of SG in people with neurodegenerative disorders in a nutshell.

**Are SG adapted to whom?**
- SG are completely adapted to older people with MCI; - Designing SG for people with dementia is challenging, but important.
**What should be the SG target?**
- Assessment, training and promoting wellbeing are good targets for people with MCI and dementia - For MCI, SG for physical and cognitive stimulation are particularly suitable; - SG choice should be personalized based on clinical assessment aiming at identifying training targets in different domains.
**Where should SG be used, and with whom?**
- SG can be employed both at home and in clinical facilities; - SG are more effective when the patient is accompanied by a caregiver/clinician; - some SG can be used alone; - Home-based training is still challenging due to technical issues.
**How often should SG be used?**
- Training frequency between two and four times a week were rated as the most adapted; But - Frequency of use for SG should be personalized based on the game features and the patient’s clinical profile and motivation; - Clinician follow up is crucial to keep the SG motivating (no loss of interest, no addiction).

### SG for Whom?

Serious Games were rated as more adapted to people with MCI compared to people with dementia. The results are converging with those reported in our previous recommendations (SG were considered between ‘adapted’ and ‘very adapted’ to people with MCI, and between ‘not very adapted’ and ‘adapted’ to people with AD and related disorders; Robert et al., 2014), and suggest that SG may be more useful for people with initial cognitive decline than to people which are already loosing autonomy in activities of daily living. This can be explained, on one hand, by the recognition that the cognitive decline associated to dementia (working memory, attention, etc.) makes it more challenging to design and employ SG in this population; and on the other hand, by the recognition that early interventions targeting initial cognitive decline are supposed to be more effective compared to late interventions (Barnett et al., 2014). Anyway, SG are considered as adaptable also to people with dementia. This is confirmed by recent studies showing that SG are usable in people with dementia both when played alone (e.g., Manera et al., 2015) and with a clinician (Ben-Sadoun et al., 2016). Ongoing studies are also exploring the efficacy of ICT-based devices (e.g., avatars, contextual helps) in supporting older adults with SG use, showing initial promising results.

### What Should Be the Target?

Assessment, training and promoting wellbeing were all rated as good targets for SG in people with MCI and dementia. Similarly, in the 2014 recommendations, assessment, stimulation and rehabilitation were all rated as good targets for people with AD and related disorders. Favoring social exchanges was not considered as the best target because most of the existing SG for older adults are not social (i.e., they are designed for a single player). However, emerging SG are also targeting the social domain. Some of these SG are showing promising results (Ben-Sadoun et al., unpublished data). Training targeting the cognitive domain and teaching contents may be more suitable to people with MCI compared to people with dementia, as they require some intact learning abilities to be optimized. However, beyond the selection of a clinical target for each patient’s category, an important aspect to take into account is that the selection of a SG should be *personalized*, and based on extensive clinical assessments aiming at identifying primary and secondary targets in the cognitive, motivational and emotional domains for each patient (Mishra et al., 2016). The assessment can also help to define the main follow up parameters, and the kind of feedback needed by each patient based on his/her challenges. For all conditions, SG should ultimately aim at targeting improvements in daily activities (autonomy). In other words, improvements in game activities should generalize to untrained abilities (Anguera et al., 2013), and demonstrate an impact on real life. SG design principles, such as inclusion of long-term goals embedded in a cohesive narrative instruction, and of specific generalization activities (e.g., instructional supports), may be important for encouraging transfer of knowledge from the computer to in-person settings. However, even improving autonomy in the SG activity alone could have a positive impact on motivation and quality of life: indeed the need of autonomy is one of the main drivers of the intrinsic motivation to play videogames in younger people (Deci and Ryan, 2000; Przybylski et al., 2010). This does not necessarily extend to people with ND. Is the need of autonomy the main motivational driver also for older adults with cognitive impairment, or are there other needs (e.g., social satisfaction/recognition)? To advance the work in this area, more research should be devoted to the design of SG in these target populations (De Schutter and Vanden Abeele, 2015).

### Where Should SG Be Used, and with Whom?

The experts suggested that most of the SG are more effective when the patient is accompanied by a trained caregiver (similarly, in the 2014 paper, SG were rated as more adapted for use with a family/professional caregiver, or a healthcare professional). This is consistent with the general recommendations on SG usability drafted by Alvarez et al. (2012) who suggested the importance of assisting the player to improve his/her game understanding (how to play?) and motivation (why to play?). The presence of a caregiver is considered as more important for people with dementia compared to people with MCI, due to their lower degree of autonomy. The presence of a trained person is important for different reasons, including: to motivate people to use the game; to help progressing in the game scenario (in case people get stuck), reminding them about game rules and commands; to make sure the SG are played safely, especially for physical SG; and to embed the SG in a positive social and emotional context. The fact that the presence of a caregiver represents a key element in SG adoption raises potential ecological and economical barriers to the use of SG outside the research and clinical context. Possible solutions include involving a family caregiver, organizing SG group sessions, or providing remote assistance. Avatars and other game assistance solutions may also be useful to promote independent SG use in people with cognitive impairment (Phan Tran et al., unpublished results).

Concerning the Where question, most of the SG are considered as useful to be delivered both at home and in clinical facilities. Clinical facilities have the advantage to allow a complete standardization of the training, and to provide a secure, controlled environment. The main problem, however, is represented by the travel time and costs. In order to improve study adherence, Ben-Sadoun et al. (2016) used a taxi to transport patients to the clinical facility, resulting in a 100% adherence to the training. This strategy was also used by Maillot et al. (2012) for some of elderly subjects which came to the clinical facility during their Exergame training study. Although this is feasible in the context of a clinical study, it would be important, in the long term, to bring SG in the patient’s home for the classical care. At present home-based training is still challenging due to technical issues (e.g., hardware setup, availability of an internet connection for data transfer), and to difficulties to monitor the training remotely, raising possible security problems. As technologies improve every day, well-designed feasibility studies in home-based setting are urgently needed to verify if home-based stimulation is safe and feasible, and for which patient populations. This is particularly relevant because new generations of older adults will be more and more used to employ ICT at home, thus potentially reducing usability problems.

### How Often Should SG Be Used?

Training frequencies between two and four times a week were rated as the most adapted for people with MCI and dementia. Evidence collected in recent studies on both cognitive and cognitive-physical training converges with this recommendation (e.g., Anguera et al., 2013; Ben-Sadoun et al., 2016). However, these frequencies should be interpreted with caution. The frequency of use for specific SG should vary depending on a number of variables, including the presence of physical activity (and its intensity), the duration of each game session, the time that patient (and eventually the caregiver) can devote to the activity, and the motivation to play. To maximize the training efficacy, it is important to establish the session frequency and duration (as well as the total training duration) based on the goals that the training is willing to achieve, and on the person’s features. For instance, if the target for a patient is to improve the general physical fitness, a short intense Exergame training is probably less adapted compared to a long training with regular sessions, in which physical activity is progressively increased based on performance improvements. But the session duration and goals need to be adapted to the person’s baseline physical fitness level, taking into account eventual physical constraints and concurrent pathologies (for instance, the training designed for an ex-marathon runner should be radically different from the training for a smoker whose previous level of active physical activity was limited).

Another crucial aspect to take into account when designing training is the motivation aspect. In general, the longer and more intense the training, the better. However, ‘forcing’ employing the SG too often may result in lowered motivation, or even in addiction, as there is a fine line between motivational and addictive aspects (Smith et al., 2011). Ideally, the features of the training should be adapted in order to increase the patient’s motivation to play, thus optimizing the training results. Strategies to improve motivation include, for instance, the presence of a clinician motivating the person, helping to set the training pace, and modifying its frequency in a timely manner based on the patient’s changes; the design of an adaptive game challenge, that keeps the player in the ‘flow zone’ (the feeling of complete and energized focus in an activity, with a high level of enjoyment and fulfillment) and improve the feeling of self-efficacy; for instance, the game becomes more difficult as the player progresses, but steps back to an easier level when the player is tired or show a decline in performance; or the presence of a well-designed game reward system.

## Limitations

The present recommendations were gathered from a relatively small group of experts working in the domain of SG for health. In further work, it would be interesting to verify if these results hold for a wider expert population (e.g., through web-survey). Furthermore, it would be interesting to collect the opinion of healthcare professionals who do not work with SG and ICT in their practice, to verify the barriers for SG adoption in the healthcare domain. For instance, if clinicians non-expert in ICT consider SG as not adapted and not useful for people with ND, they will hardly suggest their use for training purposes. This means that more effort should be made to share the promising results of SG in these populations among the clinical community.

## Author Contributions

VM analyzed the data. All the authors contributed to draft the paper.

## Conflict of Interest Statement

AG is co-founder, shareholder, BOD member, and advisor for Akili Interactive Labs, a company that produces therapeutic video games. PF works for Genious group, a company that produces serious games. All the other authors declare that the research was conducted in the absence of any commercial or financial relationships that could be construed as a potential conflict of interest.
